# Neoliberalism and precarious work in nursing in the COVID-19 pandemic: repercussions on mental health

**DOI:** 10.1590/1980-220X-REEUSP-2021-0257

**Published:** 2022-01-14

**Authors:** Larissa de Almeida Rezio, Elda de Oliveira, Aline Macêdo Queiroz, Anderson Reis de Sousa, Sonia Regina Zerbetto, Priscila Maria Marcheti, Cíntia Nasi, Maria do Perpétuo S. S. Nóbrega

**Affiliations:** 1 Universidade Federal do Mato Grosso, Cuiabá, MT, Brazil.; 2 Universidade Federal de São Paulo, São Paulo, SP, Brazil.; 3 Universidade Federal do Pará, Belém, PA, Brazil.; 4 Universidade Federal da Bahia, Salvador, BA, Brazil.; 5 Universidade Federal de São Carlos, São Carlos, SP, Brazil.; 6 Universidade Federal de Mato Grosso do Sul, Campo Grande, MS, Brazil.; 7 Universidade Federal do Rio Grande do Sul, Salvador, BA, Brazil.; 8 Universidade de São Paulo, Escola de enfermagem, São Paulo, SP, Brazil.

**Keywords:** Pandemics, Work, Mental Health, Nursing, Capitalism, Pandemias, Trabajo, Salud Mental, Enfermería, Capitalismo, Pandemias, Trabalho, Saúde Mental, Enfermagem, Capitalismo

## Abstract

**Objective:**

to understand how the contradictions and tensions of neoliberal policy, materialized in precarious work, affect nursing workers’ mental health in the context of the COVID-19 pandemic.

**Method:**

this is a study with a qualitative and descriptive approach, analyzed in the light of neoliberal economic policy. Data were collected through virtual means, with the participation of 719 nursing workers, from April to June 2020. To organize the data, the IRaMuTeQ^®^ software and thematic analysis were used.

**Results:**

the reports revealed the lack of value of workers and the loss of social labor rights; the progressive nature of the neoliberal policy, its threats and repercussions on workers’ mental health; and recognition by female workers that political and class participation does not occur in isolation, but collectively.

**Conclusion:**

under the aegis of neoliberal policy, the COVID-19 pandemic brought an upsurge precarious work, influencing nursing workers’ subjectivity and mental health.

## RESUMO

**Objetivo:**

compreender como as contradições e tensões da política neoliberal, materializadas na precarização do trabalho, repercutem na saúde mental das trabalhadoras da enfermagem no contexto da pandemia de COVID-19.

**Método:**

trata-se de estudo de abordagem qualitativa e descritiva, analisado à luz da política econômica neoliberal. Dados coletados por meios virtuais, com participação de 719 trabalhadoras de enfermagem, no período de abril a junho de 2020. Para a organização dos dados, foi utilizado o *software* IRaMuTeQ^®^ e análise temática.

**Resultados:**

os relatos revelaram o desvalor das trabalhadoras e as perdas dos direitos sociais trabalhistas; a progressividade da política neoliberal, suas ameaças e repercussão na saúde mental das trabalhadoras; e reconhecimento das trabalhadoras de que a participação política e de classe não ocorre de forma isolada, mas coletiva.

**Conclusão:**

sob a égide da política neoliberal, a pandemia de COVID-19 trouxe um recrudescimento da precariedade do trabalho, influenciando na subjetividade e na saúde mental das trabalhadoras de enfermagem.

## RESUMEN

**Objetivo:**

comprender cómo las contradicciones y tensiones de la política neoliberal, materializadas en la precariedad del trabajo, afectan la salud mental de los trabajadores de enfermería en el contexto de la pandemia de COVID-19.

**Método:**

se trata de un estudio con enfoque cualitativo y descriptivo, analizado a la luz de la política económica neoliberal. Datos recolectados a través de medios virtuales, con la participación de 719 trabajadores de enfermería, de abril a junio de 2020. Para organizar los datos se utilizó el software IRaMuTeQ^®^ y análisis temático.

**Resultados:**

los informes revelaron la desvalorización de los trabajadores y la pérdida de los derechos sociolaborales; el carácter progresivo de la política neoliberal, sus amenazas y repercusiones en la salud mental de los trabajadores; y el reconocimiento por parte de las trabajadoras de que la participación política y de clase no se da de forma aislada, sino de forma colectiva.

**Conclusión:**

bajo la égida de la política neoliberal, la pandemia de COVID-19 provocó un recrudecimiento de la precariedad laboral, incidiendo en la subjetividad y la salud mental de los trabajadores de enfermería.

## INTRODUCTION

In Western societies, public health systems and forms of work organization were negatively impacted by capitalism and the arrival of neoliberal politics. Neoliberalism is not static and ahistorical; its origin comes from the need for capitalist accumulation and difficulties-decrease in the rate of profit and social struggles. The first manifestations were privatizations, deregulation of labor and market relations, fiscal and monetary adjustment^(1)^. In this context, there is the neoliberal State, which changes labor relations, proposes changes in legislation and in productive restructuring^(2)^.

Neoliberalism, as a theory of political-economic practices, is configured in the logic of individual entrepreneurial freedoms and capacities^(3)^, reverberating in a permanent process of changes in working conditions, based on solid rights to private property, free market and free trade^(3)^.

The neoliberal policy imposes a mode of production that reconfigures the organization of institutions and work techniques, such as making labor norms/rules more flexible under market demand, streamlining the performance of activities, reduce costs, intensify work and reduce wages^(1,2)^, which promotes an imbalance between supply and demand for labor, resulting in structural unemployment, outsourced work and workforce devaluation^(4,5)^. This process can be understood as conditions that undermine labor relations^(3,6)^.

In Brazil, the neoliberal policy materializes in the health system, degrades social public policies and weakens the Unified Health System (SUS – *Sistema Único de Saúde*), due to the reduction in public investments and expenditure and precarious work^(2,7,8)^. With the current economic crises and within the scope of work in health and the nursing profession, neoliberalism has become more accentuated and triggered the outsourcing of care, the constant dismissals caused by precarious and unprotected ties, overload and high worker turnover, deconstruction and/or destruction of professional identity, causing absenteeism, absence and illness at/through work^(9)^.

This expropriation of social rights inherent to neoliberalism, as well as changes caused by financial globalization and new forms of management, negatively impact workers’ subjectivity, well-being, the way they work and the way they organize themselves collectively^(10,11)^. In nursing, this logic affects work accidents, physical illness and mental distress^(4)^, which are manifested in somatic symptoms, sleep disorders, psychoactive substance abuse, anxiety and depression, which even affect quality of care^(2,9)^.

The Coronavirus Disease (COVID-19) pandemic, which started at the end of 2019, caused by the new coronavirus (SARS-CoV-2), has been requiring public health measures and intensive care^(12)^ facing more than two hundred and fifty million infected and more than five million deaths by November 2021 in the world^(13)^. This historical fact accentuates the vulnerability of the health system and the precarious working conditions of nursing, which were already impacted by the neoliberal policy.

Thus, in order to broaden the debate about the lack of working conditions within this profession in the context of the COVID-19 pandemic, the following question was asked: how do contradictions and tensions of neoliberal policy, materialized in precarious work, affect nursing workers’ mental health in the context of the COVID-19 pandemic? This study aimed to understand how the contradictions and tensions of neoliberal policy, materialized in precarious work, affect nursing workers’ mental health in the context of the COVID-19 pandemic.

## METHOD

### Type of Study

To access the knowledge, perceptions and worldviews of nursing workers working in different health care settings, on the contradictions and tensions of neoliberal policy and its repercussions on mental health, in the context of the COVID-19 pandemic, it was considered appropriate to do so through a qualitative and descriptive approach^(14)^. The Consolidated criteria for Reporting Qualitative research were used (COREQ)^(15)^.

### Theoretical-Conceptual Framework

Work is essential for the process of sociability, which involves human relationships, and social reproduction, which is made up of ways of working and living^(4)^. It is impossible to separate the subject, the environment and the historicity of the social contexts where work takes place in the capitalist world, as the way of working can generate wear and tear or harm to workers’ health^(16)^. Currently, neoliberal policies have impacted workers’ subjectivity and mental health, since subjectivity is one of the internal processes of man, socially constructed and intersected by objective and concrete processes of sociability in everyday life^(17)^.

The nursing work process is anchored in the organization of society and human relations^(18)^, and its object of work is chosen based on health needs, in which care is conducted through tools and/or practice instruments^(19)^, anchoring itself in the execution of divided, repetitive, measured activities and in the relationship between workers and subjects-cared for^(18)^. In this process, a tensioned, reactive flow is established, which produces overload, work intensity, imposing on workers objective pressure, related to the demand for activities, and a subjective pressure, related to the responsibility of care management^(18)^. Moreover, it causes physical and emotional problems in workers^(20)^, generating insecurity.

In the logic of capitalism, health and provision of health care are treated as goods, and those who receive care are considered consumers, clients^(10)^. That said, the analysis of work in health, particularly nursing, in the context of COVID-19, is supported by the neoliberal productive restructuring, which organizes work based on quantitative productivity and social rights expropriation^(17,20)^.

### Local

Considering the country’s territorial dimension and the measures of social distancing imposed by the need to control the dissemination of COVID-19, this research was carried out in a virtual environment in all regions of Brazil.

### Participants

The invitation to participate in the survey was carried out through social networks (Facebook, Instagram, Twitter and WhatsApp), in which the snowball technique was adopted, which uses reference chains^(21)^. The study included 719 nursing professionals, living in the five regions of Brazil.

Nurses, midwives, technicians and nursing assistants, of any nationality, working in different health care settings (direct and/or administrative/managerial assistance), teaching and research, or not currently working (unemployed/retired and/or away) were included. Professionals outside the country during the data collection period were included.

There was a majority participation of females. These were identified by the initial “Nur_Work”, Nursing Worker, and the number referring to the sequential order of participation in the research.

### Data Collection

Data collection for the survey took place from April 22 to June 8, 2020, using Google Forms®, consisting of multiple choice questions on sociodemographic, labor and health data (pre-existing diseases; psychological support; psychiatric treatment; use of psychotropic drugs before and during the pandemic; previous mental distress; lifestyle habits; consumption of alcohol and other drugs; psychological support from the institution that works or studies; adoption of therapeutic and self-care measures) and the open-ended question “describe your experiences and feelings throughout the context of the COVID-19 pandemic, whose answers were analyzed in this study”. To guarantee reliability in the collection, the incomplete or semi-completed forms were eliminated and the last one sent by the participant was kept. Data extraction and organization were carried out by four researchers, together, online.

### Data Analysis

The collected data were extracted, coded and grouped into a single text file corpus. The IRaMuTeQ^®^ software was used (*Interface de* R *pour les Analyses Multidimensionnelles de Textes et de Questionnaires*) 0.6 alpha 3^(22)^. The program made it possible to carry out five types of analyzes on the text as a whole: classic textual statistics; research on specific groups; descending hierarchical classification; similarity analyses; and word cloud^(22)^. Text set processing in IRaMuTeQ^®^ with descending hierarchical classification offered four classes of text segments without coding, *a priori*.

This article referred to the second class of the result of IRaMuTeQ^®^ analysis, called “work (de)valuation”, in which saturation occurred with 216 elementary context units (ECU), which corresponded to 17.5% of the corpus total. After processing the data by IRaMuTeQ^®^, all responses were read in full. Thematic analysis was applied to the results derived from the lexical analysis of the text segments, which, based on the speeches, composed the corpus of analysis with the following steps: familiarization with the data through exhaustive readings and re-readings; coding and searching for relevant topics in response to the research question; and interpretation phase.

### Ethical Aspects

The study followed the Resolution of the Brazilian National Health Council (*Conselho Nacional de Saúde*), approved by the Brazilian National Research Ethics Commission (CONEP – *Comissão Nacional de Ética em Pesquisa*), on April 4, 2020, (Opinion 3,954,557/2020). There was consent through an online Informed Consent Term. Autonomy, right to withdraw, confidentiality and data protection were guaranteed to them.

## RESULTS

A total of 626 (87.1%) women participated, with an average age of 25 to 45 years, from the Southeast (232; 32.3%), Northeast (193; 26.8%), North (129; 17.9%), Midwest (99; 13.8%), and South (66; 9.2%). As for marital status: married (292; 40.6%), single (265; 36.9%), stable union (102; 14.2%), divorced (47; 6.5%), separated (11; 1.5%) and widow (02; 0.3%). As for race/color: white (343; 47.7%), brown (277; 38.5%), black (78; 10.8%), yellow (14; 1.9%), indigenous (05; 0.7%), others (02; 0.3%). As for professional category: nurses (79.9%), technicians (16.4%) and nursing assistants (3.7%), of these, 65.6% worked in direct care, 72% in services public, with an average professional training time of 14 years.

The themes derived from the reports inform the productive restructuring, which has neoliberalism as one of its components, which affects not only nursing work, but the lives of all female workers^(19)^.

### The Disvalue of Female Workers and the Loss of Social Labor Rights

During the pandemic period, nursing workers face exhausting work, with overload and increased time for activities, with few rest conditions when working for more than eight hours. The criticisms expressed show problems prior to the pandemic, however intensified at the present time.

*It is being an extremely exhausting work, hospitals accept patients referred by the regulation, but they do not have enough nursing staff, making our work exhausting and precarious. (Nur_Work_405); there is a work overload for nursing professionals and very few rest conditions during work breaks of more than eight hours. (Nur_Work_686)*

Nursing workers, who are on the front line of the pandemic, point to the absence of minimum conditions to carry out their activities, such as the lack of Personal Protective Equipment (PPE), low wages and worker devaluation.

*Working on the front lines of this pandemic has not been easy, we are less valued every day, we do not have the ideal equipment to develop our work, we do not have a deserved wage, our nightly pay is minimal and the workload is enormous. (Nur_Work_29); we don’t have equipment and we have to work with exhaustive hours. (Nur_Work_231)*

They announce the loss of social labor rights, problematize the reduction of stable jobs, the privatization of state institutions, and the outsourcing of work contracts. They also highlight the weakening and dismantling of the Brazilian health system, with political actions that affect the population’s living conditions and health.

*The city’s municipal management is trying to fire employees, to institute an outsourced service, with disregard and disrespect for public servants (Nur_Work_639); took our vacation and are wanting to take the Annual Christmas Bonus Wage, every day, they are increasing our responsibilities and making more demands. (Nur_Work_513); we know the importance of public health and of some political measures that, for some time, have been weakened and deconstructed in favor of retrograde and meaningless ideals. (Nur_Work_214)*

In view of the exposed reality, nursing workers point out that there is tension between the need to work for the duty of professional practice and fears of the risk of exposing their own health in the face of precarious working conditions. There are conflicting feelings regarding the need to work and take care of people, and the desire to protect themselves or not to remain in nursing. Confronted with this reality, they express anguish and fear before the precarious work:

*We do our work because we understand the needs of the population, but, in fact, the conditions are precarious, this causes me anguish and sometimes I think about changing my profession. (Nur_Work_378); I wish I could choose to protect myself or work. I leave home every day not wanting to go out. I’m very scared of what’s still going to happen. I wish I could stay home. (Nur_Work_17)*

### The Progressive Nature of Neoliberal Policy, Its Threats in the Context of the Covid-19 Pandemic and the Repercussions on Female Workers’ Mental Health

Nursing workers point to a reduction in the State’s presence in guaranteeing the category’s labor rights. These situations create insecurity at work and the emergence of feelings linked to precarious working conditions:

*I would really, really want these politicians and public managers to look more at our nursing class, as we are face to face with all this chaos and we are underpaid and unrecognized for our work. (Nur_Work_331); the misgovernance and debauchery of the President of the Republic, among other things, have allowed us to be experiencing several feelings at the same time. (Nur_Work_588)*

Due to the lack of human resources, nursing workers were relocated from their work sectors to those that care for people affected by COVID-19, changing their routine and resulting in work overload. Added to this, the imminent risk of contamination and the lack of PPE aroused in them feelings of fear, anguish, which have led them to mental illness/distress and the consumption of medication to deal with this adverse situation and also with death:

*Lack of PPE, low wages, the risk of contamination is imminent (Nur_Work_06). The management decision to close my service for the relocation of professionals to direct care to COVID-19 patients has left me very anguished. (Nur_Work_37); what has been bothering us is the shortage of professionals and the overload of those who are working. Many are getting sick because they are overloaded with work. (Nur_Work_61); I work hard every day, I take two pills of natural tranquilizer every day and then I take it (…) I have already had a colleague who died, I’m very scared. (Nur_Work_67)*

The reports point to disregard for nursing workers’ life and social rights in relation to work, justified by the perception that they are considered only a number in the absence statistics. They also reinforce nursing’s position as the most affected in relation to others in the health area, because they work in an unknown situation, resulting in fear and sadness.

*I’ve been away from work for four days, unfortunately, I’m more of a stat. (Nur_Work_47); I feel fear and sadness for the devaluation of nursing, even in its importance to be in evidence, I see the disregard for the lives and rights of professionals who are treated with great negligence. (Nur_Work_537); I feel sad to know that nursing is the profession that suffers most from everything that happens; we are the front line. (Nur_Work_231)*

Nursing workers lament and suffer the death of co-workers’, mainly due to the lack of minimum working conditions. They emphasize that distress is intensified by the fact that these colleagues are not alive to witness and experience the rights that the category claims today, such as average wage and reduction of weekly workload to 30 hours.

*I feel sad for the nursing profession, which is suffering so much from the pandemic, and for being the first to suffer (…) it has been sad to lose your friends and professional colleagues due to the lack of Personal Protective Equipment. (Nur_Work_692); many of us nursing professionals have gone, unfortunately, and have not had the chance to be given the long-awaited improvements and the professional recognition and dignified wage that we are seeking so much, as well as a reduction in the workload to 30 hours. (Nur_Work_512)*

### Weakening of Class Entities and the Need to Join the Category

Nursing workers emphasize that regulatory bodies to exercise the profession and union movements have not been fighting for the needs of nursing, and the weakening of these entities seems to have been accentuated at the time of the pandemic, strengthening the feeling of worthlessness, abandonment and neglect:

*We are abandoned or neglected by our professional bodies, the Federal Council of Nursing and the Regional Council of Nursing, which show little interest in actually helping us in this moment of pandemic. (Nur_Work_503); I feel devalued as a class (…) the scrapping of public services and the lack of even the struggle of our own union and council for a minimum wage. (Nur_Work_201)*

On the other hand, the contradictions that emerge from the organization of nursing work allow to recognize that its relationship with work does not occur in isolation, but collectively, in the political and class struggle, which materializes in expressions of concern with the political engagement. However, answers that clarify a more direct and active relationship of the category with union class movements were not found:

*Nursing professionals must unite more and fight for their rights, because, at a time like this pandemic, we have seen how much we are undervalued in terms of wages and work (…) only with our union and collaboration will we be able to achieve changes and recognition. (Nur_Work_270); I believe that, at this very difficult time, empathy and union to discover new ways of working are fundamental and will certainly change our way of providing assistance, research and management. (Nur_Work_128)*

## DISCUSSION

The analysis of results reinforces the influence of neoliberalism in Brazil that, even before the pandemic, it followed a cycle of setbacks in social labor rights, which removed workers’ guaranteed rights and changed retirement rules for a significant portion of the population. In this way, it showed that the neoliberal logic in the organization of work in health produces wear and tear and illness in nursing workers.

The State, upon assuming the neoliberal policy, established a ceiling on federal public spending through Amendment 95/2016, which aggravated the underfunding of SUS for the next 20 years, limiting investments necessary for the maintenance and advancement of the system and public services. Furthermore, it restricted investment in research, in the improvement of working conditions and in the increase in the number of female workers through public examinations^(23)^.

As a result, the disvalue of nursing workers and the loss of their social labor rights were accentuated, which are expressed in this study in work overload and intensity, low wages, high turnover, shortage of human resources and inputs, non-supply of appropriate PPE and in sufficient quantity. The neoliberal logic of work organization brings disastrous consequences to workers’ mental health, supporting the literature^(2)^.

It emphasizes, however, that precarious work precedes the pandemic context, and it is revealed and also intensifies^(24)^ the fear of contamination and mental distress/illness, which worsens when the health service does not offer psycho-emotional assistance. Furthermore, it is related to one of the impacts of the capitalist mode of production, which degrades the well-being and affects nursing workers’ health in the expression of anguish, fear, medication intake and the feeling of worthlessness^(25)^, since the priority is the final product of work and not health^(6)^.

It is important to highlight that the progression of the neoliberal policy on work expands to daily life and affects workers’ subjectivity and objectivity, such as absenteeism, absence and illness at/through work^(24,25)^. In this study, these women feel just a number, de-personified as subject-nursing workers.

As workers are exposed, they have disorders such as Burnout Syndrome, depression, suicide, abuse of psychoactive substances, anxiety conditions, somatic symptoms, work stress, fatigue, derived from the flexibilization production process conditions^(2,9)^. These repercussions on mental health lead to the loss of subjectivity, singularity, work intentions, pleasure, restricting work to a means of survival, not integrated to life, but alienating. Therefore, nursing workers’ mental health-illness process, even in the context of COVID-19, is anchored in social relationships, historicity and culture that are commonly subordinated to the biological aspect.

The aforementioned problems are also related to high human disposability linked to capitalism^(4)^, in which precarious work spreads through nursing workers’ daily life, given the withdrawal of their social rights and, often, the right to life, which brings permanent uncertainties and insecurities, such as the absence of the possibility of prospecting a future due to work volatility. Furthermore, the short rest period and the non-reduction in working hours take up almost all workers’ time, impoverishing them in their subjectivity and devaluing them^(17)^.

It is noticed that the neoliberal logic and the precarious work, before and during the pandemic, brought repercussions to mental health, but also the need for articulation, the demand for support from class entities and union movements. They awakened workers, as a workforce, to the urgent need for cohesion, mobilization and political participation in the struggle for social emancipation, for a process of union and sociability in fields of practice, for an emancipatory policy for the professional category. Considering that this process is a constituent category of the social being and for the strengthening of social participation movements^(26)^, the ideal of spaces is a condition to fight for better working and living conditions^(27)^.

In this sense, the data in this study show that these spaces are distant, or could not be recognized as legitimate for nursing workers, due to the technical dimension valorization in relation to the policy in the heart of professional activity, which, combined with individualism, characteristic of neoliberal labor relations, weakens unions, associations and representatives of professional bodies, enabling greater exploitation of capital and managerial autonomy to the detriment of workers’ political participation^(27)^. However, there are still problems in the health scenario that are evident in the low political capillarity^(6)^.

A study with nursing professionals who occupied leadership positions in professional associations points out that, despite the knowledge about precarious working conditions, depoliticization, accommodation and social apathy drive nursing workers to transfer the responsibility of their struggles to the entities^(27)^. This disinterest and distancing are justified by the profession’s historical-social origin, the political and social symbolic participation of women, intensified by the historical- cultural heritage^(27)^.

The consequences and difficulties of this political reorganization impede the visibility of labor needs and precariousness, nursing workers’ social performance, a critical view of eminent power relations in the social and work context, legal changes and improvement in working conditions.

An alternative to overcome this situation is to intensify critical reflection on the characteristics and work processes of the category both in academic training and in daily work. This is possible when dialogic and emancipatory spaces are built that make it possible to analyze and identify alternatives that overcome imitative and repetitive practice^(28)^.

Finally, it is noteworthy that nursing professionals who are on the front line of care are the group most exposed to COVID-19. However, it is not homogeneous, even exposed in the work environment, mental health is affected in a unique way. On the other hand, it is essential to consider the power relations between professional, gender, social class and race/color categories necessary to broaden the debate on the repercussions on the lives of these nursing workers about the “new from COVID-19”^(29)^.

The limitations of this study are that it was conducted in Brazilian regional contexts with different conditions of resources, work, types of management and employment relationships of these workers, in addition to being carried out online, which made it impossible to deepen the object of investigation.

### Contributions to Nursing and Health

This study contributes to advancing the debate on the repercussions on nursing workers’ mental health in light of precarious work, intensified in the context of the COVID-19 pandemic. The research also reveals the need for mental health care, regardless of the pandemic moment, as they were previously exposed. It can support the discussion and mobilization of nursing workers for inclusion in a collective struggle for better working conditions. It guides the need to strengthen unions and associations, to implement and create social protection policies, which have been lost and/or weakened in the neoliberal agenda. Problems faced by workers require the union of class and representative bodies, to face the neoliberal policy not restricted to nursing, but to the world of work.

## CONCLUSION

It was understood that the progressive nature of the neoliberal policy imposes the disvalue of nursing workers, takes away their social rights, affects their subjectivity and mental health and extends to their daily lives.

There is an increase in precarious work, in this pandemic moment, in terms of scarcity and/or non-supply of PPE, in the reduction of nursing workers, in the physical and emotional overload. There was an intensity of nursing work, due to the large number of hospitalized people, the absence of psycho-emotional support by institutions and that such situations expose work disvalue and the risk to nursing workers’ lives.

The progressive nature of the neoliberal policy, in the context of the COVID-19 pandemic, reveals the contradiction in this scenario, in which, instead of improvements in working conditions, nursing workers face intense precariousness in the health work world. This contradiction impacts mental health, resulting in psychological distress, expressed by feelings of sadness, fear, irritability and anguish in the face of precarious work conditions. On the other hand, this distress mobilizes the category to reflect on the needs for social participation and politicization of nursing.

It was revealed that nursing workers express work as a central category in their lives, as they could not choose between working or not working and maintaining their lives. It should be noted, however, that this need is not restricted to nursing, but to all female workers, above all requiring the overcoming of the social individualization of work and female workers. Since the pandemic situation exposes everyone to precarious world of work, it highlights the fragility and/or absence of a social protection system that goes far beyond nursing work.

## Financial support

This study was financed by the Conselho Nacional de Desenvolvimento Científico e Tecnológico – CNPq, Ministério da Ciência, Tecnologia, Inovações e Comunicações – MCTIC, Ministério da Saúde – MS. MCTIC/CNPq/FNDCT/MS/SCTIE/Call Decit 07/2020.
